# AI-Enhanced Qualitative Analysis in Healthcare: Unlocking Insight from Interviews of Leadership at Top-Performing Academic Medical Centers

**DOI:** 10.3390/healthcare14020248

**Published:** 2026-01-19

**Authors:** Triss Ashton, Seth Chatfield

**Affiliations:** 1 Department of Management, Tarleton State University, Stephenville, TX 76401, USA; 2 School of Business, Shenandoah University, Winchester, VA 22601, USA; schatfie@su.edu

**Keywords:** Large Language Models (LLMs), artificial intelligence in healthcare, qualitative data analysis, healthcare quality, text mining, Donabedian model

## Abstract

**Highlights:**

**What are the main findings?**

- Freely available large language models (LLM) rapidly analyzed textual healthcare data yielding a 10-factor, 24-subtopic structure consistent with traditional manual LSA results.
- AI systems identified theoretical linkages with the Donabedian model of healthcare quality that traditional manual analysis had missed.

**What are the implications of the main findings?**

- LLM-based analysis can reduce the time, expertise, and labor required to extract meaningful insights from large qualitative datasets in healthcare settings and potentially uncover deeper insights than traditional methods.
- Freely available AI tools increase the accessibility of text-based analytics, enabling healthcare organizations to extract meaningful operational and patient quality insights from existing qualitative data.

**Abstract:**

**Background/Objectives**: Vast amounts of textual data are generated by healthcare organizations every year. Traditional content analysis is time-intensive, error-prone, and potentially biased. This study demonstrates how freely available large language model (LLM) artificial intelligence (AI) tools can efficiently and effectively analyze qualitative healthcare data and uncover insights missed by traditional manual analysis. Interview data from chief nursing officers (CNOs) at top-performing academic medical centers were analyzed to identify factors contributing to their operational and patient quality success. **Methods**: Semi-structured interviews were conducted with CNOs from top-performing academic medical centers that achieved top-decile quality measures while using resources most efficiently. Interview transcripts were analyzed using a mix of traditional text mining in LSA and Gemini 2.5. The capability of four freely available AI platforms—Gemini 2.5, Scholar AI 5.1, Copilot’s Chat, and Claude’s Sonnet 4.5—was also reviewed. **Results**: LLM AI analysis identified ten primary factors, comprising twenty-four subtopics, that characterized successful hospital performance. Notably, AI analysis identified a theoretical connection that manual analysis had missed, revealing how the identified framework aligned with Donabedian’s seminal structure, process, outcomes quality model. The AI analysis reduced the required time from weeks to nearly instantaneous. **Conclusions**: LLM AI tools offer a transformative approach to unlocking insight from the analysis of qualitative textual data in healthcare settings. These tools can provide rapid insight that is accessible to personnel with minimal text-mining expertise and offer a practical solution for healthcare organizations to unlock insight hidden in the vast amounts of textual data they hold.

## 1. Introduction

Healthcare, like other businesses, has increasingly adopted computers in clinical settings [1] and in administrative environments. Like other businesses, increased computer use leads to more textual data in databases. Text data is unstructured, ambiguous, and difficult to process [2]. A textual dataset, referred to as a corpus, can be generated by any number of processes. For instance, studies of patient satisfaction based on interviews [3], clinical notes for testing drug safety [4], patterns in off-label drug use [5], pharmacovigilance [6], and patient diaries [7] are examples of text data in the medical field. These datasets and others hold tremendous amounts of knowledge that are there for the taking to enhance the quality of care and expand scientific knowledge and biomedical understanding.

However, classically, such data are analyzed manually by teams of analysts using methods such as content analysis. It can take weeks to analyze a corpus by content analysis, and the resulting analysis may be biased [8]. Text mining is a much faster, free from human bias, computerized alternative [9]; however, even text mining has its limits. In a typical text-mining analysis, where a computer-based algorithm breaks the data into theme-based clusters in a process very similar to factor analysis, defining the clusters returns to a manual process similar to content analysis. Where the discovery of the clusters, referred to as topics or factors, is an efficient process time-wise, determining the identity or subject of the clusters is time-inefficient.

Large language models (LLMs) in artificial intelligence (AI) introduce a phenomenal alternative. For instance, in 2024, Microsoft researchers introduced a new two-phase text analysis framework called TnT-LLM (Taxonomy Generation and Text Classification with an LLM in both phases) [10]. In TnT-LLM, phase 1 is a taxonomy generator that categorizes or clusters the data, similar to the text mining effort described above. Then, in phase 2, the LLM produces a text classification or describes the clusters, similar to the previously mentioned cluster-defining process [10].

Many of the LLMs available today self-report that they can perform a variety of analyses on text data, using many or most of the known text mining methods. During chat sessions, those AI systems reviewed in the literature review describe or propose analysis strategies that generally follow the TnT-LLM framework.

In recent years (2020–2025), many studies have examined the use of AI in healthcare; however, of the studies sampled, the researchers continued to use traditional qualitative analysis methods, such as thematic analysis, reflexive thematic analysis, grounded theory, and meta-synthesis, to analyze interview data [11,12,13,14,15,16]. Several of these papers cite [17] as the methodological authority for conducting thematic analysis. Outside of healthcare, researchers have recently begun studying how to apply AI to qualitative analysis; however, that work is primarily focused on methodological development. This literature is relatively extensive; here, we cite only a small sample [18,19,20,21]. In contrast, this study may be the first to apply AI to qualitative healthcare data, with the overarching goal of developing new knowledge.

To study the applications and benefits of LLMs in healthcare, we examined data collected from a study of teaching hospitals at some of the nation’s most successful academic medical centers. These hospitals were sampled from a population of Council of Teaching Hospital (COTH) members (*n* = 277) based on data envelopment analysis scores, identifying hospitals that most efficiently converted inputs into outputs. Outputs measured included case-mix-adjusted discharges, outpatient visits, teaching intensity, and value-based purchasing scores (process of care, HCAHPS, and mortality). Inputs measured included labor, capital, hospital beds, and service complexity. That study generated a corpus composed of interview responses from chief nursing officers [22,23]. The objective of the interviews was to determine, based on self-reporting, what operational aspects made these medical centers so successful.

To assess whether sample hospitals (identified as DEA-efficient) differed financially from other COTH member hospitals, we compared their five-year mean operating margins. The sample hospitals averaged 2.56% compared to −10.42% for the remaining population of COTH. An independent-samples *t*-test with unequal variances was conducted to determine statistical significance in the mean operating margin between the population and the sampled hospitals. There was no homogeneity of variances in the five-year mean operating margin between the sampled and non-sampled hospitals. Equality of variance was assessed by the F-test for equality of variances (a parametric test) and Moses test (a non-parametric test) [24]. Both tests indicated a statistically significant difference in the variances of the two samples, with *p* < 0.0001 for the F-test and *p* = 0.0033 for the Moses test. Finally, a *t*-test comparing the mean operating margin between the selected hospitals and the non-selected group found a significant difference (*p* = 0.0102). This result suggests that the sample hospitals not only outperformed on all DEA output measures (case-mix-adjusted discharges, outpatient visits, teaching intensity, and VBP process of care, HCAHPS, and mortality), but also on operating margin.

This research contributes in two ways. First, given the vast amount of data locked up in corpora across the medical community, we review a method of analysis using LLMs that employees/analysts with minimal text-mining knowledge can replicate. Such persons may be found in the IT departments of most medical centers, or, if the center is fortunate, in the analytics center. Second, from the interview data, an unexpected variant of the Donabedian model emerged, comprising 10 factors and 24 subtopics. This model essentially defines how successful medical centers operate on a daily basis. However, it is not a linear process that proceeds from structure → process → outcome, as the Donabedian is generally described. Instead, it is a cyclical systems model, composed of several interrelated pieces.

## 2. Literature Review

### 2.1. Quality Models in Healthcare

The Donabedian model serves as a seminal instrument in the understanding and evaluation of healthcare quality and is based on three primary attributes of any health services organization: (1) structure, (2) process, and (3) outcome. In the 2005 reprint of his influential 1966 article, Avedis Donabedian explores the three interconnected concepts of structure, process, and outcomes as a composite whole [25]. Despite being introduced more than five decades ago, Donabedian’s model remains a lasting and influential framework for the delivery and measurement of healthcare quality [26,27].

According to Donabedian, *structure* concerns the adequacy of the facility and equipment, the organization’s structure, the operation of the systems providing care, the organization’s financial structure, and the qualifications of the clinical staff [25]. *Process* examines the steps of care, including the completeness of the diagnosis, the technical competence in performing a medical procedure, and the continuity of care provided to the patient. Essentially, the process is asking the question, “has what is now known to be ‘good’ medical care been applied” [25] (p. 694). *Outcomes* represent the effects of care on health status. The results. Outcomes can encompass numerous measures, alone or in a composite, that represent the effectiveness of care as defined by individual members of a particular society or culture. Examples include clinical outcomes, patient satisfaction, or various other quality metrics [25].

Donabedian’s framework has and continues to be used in the field of healthcare quality improvement from the system level to individual facilities. In 2007, the Institute for Healthcare Improvement proposed 13 non-disease-specific measures addressing structure, process, and outcomes of care for system-level quality indicators [28]. The Donabedian model is also used prospectively as a planning framework; for instance, their case study examines how White Plains Hospital developed its response to the COVID-19 pandemic during the initial wave of 2020 [29]. Additionally, the Centers for Medicare & Medicaid Services (CMS) has used the Donabedian model as a key framework for developing and assessing quality measures, with most measures designated as structure, process, or outcome to evaluate and compare the quality of healthcare organizations [30].

This framework is particularly significant for academic medical centers with their complex structures, multifaceted care processes, and wide-ranging outcome measures. Gaining insight into how chief nursing officers in top-performing academic medical centers think about organizational effectiveness in terms of patient quality helps reveal how the linked and interrelated dimensions of structure, process, and outcomes converge in successful healthcare institutions

### 2.2. Text Mining—Introduction

Text mining attempts to extract meaningful information by identifying factors or clusters of documents with similar subjects, as reflected in word choice [31]. Its use is continually expanding, and it is increasingly used in healthcare research. For instance, ref. [32] used it to study government communications and supply-demand dynamics; in another study, text mining was used to study tweets related to diet, diabetes, exercise, and obesity [33]. There are several supervised and unsupervised methods for performing text mining [34]. Unsupervised methods are more ideal for discovering hidden structures [35]. The algorithms in the unsupervised class have similar processing steps and about the same level of performance [9]. However, data issues or characteristics can cause one algorithm to perform better in select instances [36]. As such, latent semantic analysis (LSA), an algebraic method similar to factor analysis, was selected to illustrate AI functionality in the analysis.

In LSA, data are prepared by cleaning and preprocessing, including eliminating punctuation, converting to lowercase, and removing common “stop words” (e.g., “the,” “is,” “and”). The data is then tokenized, i.e., split into individual words or “tokens”. The data is then converted into a numerical format, typically a word-by-document frequency matrix, to which the machine-learning algorithms are applied. The data is next vectorized using techniques like Bag-of-Words (BoW) or Term Frequency–Inverse Document Frequency (*tf-idf*) to create numerical representations of the text. At this point, any unsupervised method can be used to partition the data into clusters. In LSA, the singular value decomposition of the input matrix decomposes the data into a set of words that define clusters and a second set of documents that correlate with those clusters. These solutions are commonly referred to as the LT (Term-solutions) and the LD (Document-correlation).

Traditionally, pre-LLM AI, once the matrix was decomposed into LT and LD solutions, the corpus analysis returned to a manual analysis mode for interpretation. Analysts read the lists of words and correlated documents, developed an interpretation, and defined the various clusters or factors discovered by the algorithm. Where the analysis had been relatively free of human bias, it is now subject to potential interpretation bias [22].

### 2.3. AI System Capabilities

There is a varying but increasing capability to perform text analysis in currently available AI products. This study considered Gemini 2.5, ChatGPT’s Scholar AI 5.1, Copilot’s Chat, and Claude Sonnet 4.5. However, we note that there are several other potential systems for an analyst to consider, including Grok, Perplexity, and SciSpace. Here, we review the capabilities observed in or reported by select AIs.

#### 2.3.1. Gemini 2.5

This study started before Gemini 3.0 became available and was run on version 2.5 instead. Gemini 2.5 is not capable of running latent semantic analysis (LSA) or latent Dirichlet Allocation (LDA); however, it can perform K-Means clustering, Hierarchical Clustering, and DBSCAN. It is also capable of handling many other steps in text mining, such as vectorizing by tf-idf transformations, determining the optimal number of factors to extract via k-means clustering, and applying customer stop-word lists. More importantly, it can guide the user through an LSA or LDA analysis, write basic Python or R code for LSA or LDA, generate procedural instructions, and, in particular, interpret the resulting clusters or factors.

Gemini 3.0, introduced in November 2025, reports that it can now perform LSA- and LDA-based text mining analysis using R or Python. A closer, yet preliminary examination suggests that Gemini 3.0 cannot run that analysis but instead continues to guide the user through the process.

#### 2.3.2. Scholar AI 5.1

ChatGPT’s Scholar AI 5.1, for instance, proposed an analysis routine consisting of Text Cleaning and Tokenization, Vectorization, Clustering using algorithms such as K-Means or Hierarchical Clustering, and Cluster Interpretation. The processes outlined by the AI are the same as those outlined in the text mining section above. Scholar AI also claims the ability to run LDA, LSA, pLSA, and NMF.

#### 2.3.3. Copilot

When asked, Copilot claims it can perform text mining using most unsupervised methods, including k-means, hierarchical clustering, LDA, non-negative matrix factorization, and LSA. However, when asked to perform an LSA with a highly specific prompt, it produced a workflow that included Python code to perform the analysis but did not complete it. Here is the prompt used

*Perform LSA of the attached file CNO_Data.csv. The data file has two columns. One identifies the document, the other has the qualitative text. Use the attached stoplistHealthExec.txt to identify words to remove from the analysis. Use Porter’s stemmer. Remove terms that exceed a sparsity level of 0.977. Use tf-idf weighting. Extract 24 factors. Normalize the matrices and use varimax rotation on the final LD and LT matrices.*

However, in another instance, a more generalized prompt caused the AI to perform a complete LDA analysis in Python and interpret the resulting clusters. The prompt for that instance read,

*Analyze the data in the attached file. Identify the clusters in the data and then define and describe the clusters.*

These outcomes with Copilot could simply be a question of which version is operationalized. The discussion above refers to the free version of Copilot. In Section 3.2, various systems are applied to validate the text mining solution. During that testing, the free version would not perform the analysis. However, during that testing, the M365 version of Copilot did run an LSA.

#### 2.3.4. Claude

Anthropic’s Claude, when asked, claims it can analyze several types of qualitative data, including interview transcripts, open-ended survey responses, documents, reports, or written content, social media posts, and customer feedback. When asked to analyze interview responses, it claimed that it “performed a manual qualitative analysis by reading through all 74 responses and identifying recurring themes, concepts, and patterns. (Session link: https://claude.ai/share/f519f52e-eb29-4a99-8b94-435d38a58d6d, accessed on 15 November 2025)” When asked to define the manual process that was applied, Claude responded with the following outline [substeps and examples it provided have been truncated]:

Step 1: Initial Familiarization (First Read-Through)

Step 2: Open Coding (Second Read-Through)

Step 3: Frequency Mapping

Step 4: Pattern Recognition and Grouping

Step 5: Cluster Definition

Step 6: Theoretical Framework Application

Step 7: Validation and Refinement

Step 8: Cross-Cutting Theme Identification

When asked directly, “*Can you perform text mining using an unsupervised method?*” it produced publishable HTML code for a tool that can perform LDA-based text mining (Claude’s unsupervised text mining tool: https://claude.ai/public/artifacts/e90cc720-5c62-46b1-b597-7fb5fac45a06 accessed on 11 January 2026).

## 3. Materials and Methods

### 3.1. Data

Data was collected under the supervision of the Institutional Review Board at the University of Toledo, protocol reference number 107628. The University IRB operates “… following basic principles in agreement with The Belmont Report, The Declaration of Helsinki, The University of Toledo’s Federal wide Assurance for Protection of Human Subjects, the “Revised Common Rule” 45 CFR 46, Subpart A: and 45 CFR 46 Subparts B, C and D, as well as FDA 21 CFR 50 and 21 CFR 56” (UT, 2025). Written informed consent was obtained from all participants prior to inclusion in the study.

The data were collected through interviews with senior executives at the teaching hospitals of some of the most successful academic medical centers [22]. These hospitals were sampled based on data envelopment analysis scores, identifying major teaching hospitals that most efficiently converted inputs into outputs. Outputs measured included case-mix-adjusted discharges, outpatient visits, teaching intensity, and value-based purchasing scores (process of care, HCAHPS, and mortality). Inputs measured included labor, capital, hospital beds, and service complexity [23]. The interviews included several questions focused on nurses and the nursing aspect of the center’s operations. The questions directed to nursing executives are reported in Table 1. The responses to those questions are the basis of the corpus studied in the analysis that follows. Those interview questions generated 593 qualitative responses from 74 respondents.

### 3.2. Text Mining

For this analysis, the authors used a manual latent semantic analysis (LSA) implemented in R using human-generated code as described by [37]. The analysis followed the procedures described by [9] and as outlined in the literature review.

The analysis utilized a custom stoplist to eliminate high-frequency, non-central terms, a sparsity level of 0.977 (sparsity is the ratio of empty cells and is used to remove extremely rare terms), *tf-idf* weighting, and varimax rotation of the final solution matrices LT and LD. In total, 24 topics were extracted, consisting of terms with factor loadings above 0.45. The roots of the terms comprising the extracted topics are reported in Table 2.

To validate the analysis, the following prompt, along with the data and the stoplist used above, was provided to ChatGPT’s Scholar AI 5.1, and Copilot with chat 4.0.

*Perform LSA of the attached file CNO_Data.csv. The data file has two columns. One identifies the document, the other has the qualitative text. Use the attached stoplistHealthExec.txt to identify words to remove from the analysis. Use Porter’s stemmer. Remove terms that exceed a sparcity level of 0.977. Use tf-idf weighting. Extract 24 factors. Normalize and varimax rotate the final LD and LT matrices.*

Copilot offered a step-by-step analysis strategy, Python code to run the analysis, and offered to interpret the results; however, it did not analyze the data. The analyst would need to install Python, run the code, and recover the analysis results matrices. However, the code the AIs generated is sound and produces a solution nearly identical to that generated manually. In contrast, M365 Copilot will perform the analysis. Similarly, ChatGPT’s Scholar AI wrote an analysis strategy and decomposed the data, yielding a nearly identical solution to the one generated with R code and reported in Table 2.

**Table 3:** Factor solution generated from the LD matrix. Colors added to components correspond with Table 2 topic colors.

	Component									
	1	2	3	4	5	6	7	8	9	10
V18	0.732									
V16	−0.577									
V21	−0.478									
V9		0.719								
V2		0.644								
V15		0.489								
V22			−0.688							
V3			0.536			0.395				
V14			0.518					0.417		
V10				0.725		−0.309				
V12				0.578						
V5				0.378		0.321				
V4					0.746					
V23					0.500					
V11					−0.480					
V19						0.780				
V17							0.686			
V7							−0.628			
V1							0.310			
V24								0.779		
V8							−0.307	−0.352		
V13									0.917	
V6				−0.314						0.731
V20										0.628

Extraction Method: Principal Component Analysis. Rotation Method: Promax with Kaiser Normalization. Rotation converged in 24 iterations.

### 3.3. Factor Analysis

The topics, as represented by the term roots in Table 2, describe very specific concepts. Many of them are describing related concepts. For instance, topic 18 discusses how public reporting of quality measures enhances transparency. Topic 21 states that leaders plan based on strategic goals and educate the staff. Topic 16 explains that quality is an organizational focus and is a cultural issue. These three topics describe leadership perspectives and can be interpreted as first-order expressions that describe a second-order latent construct. They are artifacts of a higher-level concept. If so, they should co-occur in the original data and, as such, be recoverable by a factor analysis method.

Mathematically, LSA uses a singular value decomposition (SVD) algorithm from linear algebra. The SVD model is A = UΣV^T^. Principal component analysis (PCA) factor analysis uses X = TPT^T^. In both cases, they perform dimensionality reduction, but the dimensions they expose are different.

In the classic dimensionality reduction in PCA, variables (scores against scales) are grouped together based on correlation because, in theory, multiple scales measuring the same idea tend to have similar scoring patterns. Dimensionality reduction reveals the latent construct structure that the scales were attempting to measure. The important thing to observe here is that the respondent scored a “scale,” where the scale is a collection of words organized into a sentence expressing an idea.

In contrast, LSA examines collections of word tokens (root terms) and groups them based on the correlations observed in their co-occurrences. LSA’s dimensional reduction reveals the co-occurring term roots that form the foundation words for reconstructing a sentence or “scale” like item.

So, both PCA and LSA are performing dimensionality reduction. LSA reduces singular words into collections that infer something analogous to a scale item or a sentence; PCA reduces scale scores, where a scale is more or less a sentence, revealing constructs.

In LSA, the SVD algorithm produces two outputs: a matrix of topic-related terms (the LT matrix) and a matrix of documents from which those words were extracted. This second solution, also called an LD matrix (Loadings for Documents), contains factor loadings for each document, with each document scored against each factor. The loading values can be interpreted as a sort of correlation coefficient against each topic. If topics 16, 18, and 21 truly measure a single overarching concept, then they should be extractable as a single factor in a factor analysis of the LD matrix.

Principal components factor analysis was applied to the LD matrix, using Promax rotation. Ten factors were extracted from the LD matrix, accounting for 50.664% of the variance. That solution is presented in Table 3.

A reorganized Table 2, presenting the factor analysis, is shown in Table 4. Table 4 will be the subject of the AI analysis that follows.

## 4. AI Analysis Results

Once the data were factorized, the lists of keywords defining the factors were submitted to Gemini to interpret and define the clusters and develop a model for future research. The key points of the chat session are reported here, but the full session is available from the link https://gemini.google.com/share/d66028e6b36a (accessed on 11 November 2025).

Given that the factors comprised varying numbers of topics, the AI analysis focused on defining the factors based on the keywords or term roots observed in Table 4. A similar analysis performed manually, the traditional analysis process, takes considerable time to complete, generally lasting several hours or days, with the analyst repeatedly reading the source documents to produce a descriptive definition. Using AI for this step of the analysis reduced the time needed to seconds.

### 4.1. Factor 1

The task of interpreting the factors was assigned to Gemini 2.5; however, all the AIs mentioned so far are equally capable. The assignment prompts followed a simple standard form: “*I have a topic that is composed of three sets of keywords from a text mining solution. Can you identify the topic’s subject*?” The corresponding list of words (term roots) was then pasted into the chat session with the prompt. The interpretation of the first factor is provided here, along with all details generated by Gemini.

Gemini’s response (for clarity, all AI prompts and responses are italicized):

*Based on the three sets of keywords, the overall subject appears to be related to Organizational Performance, Strategy, and Governance within a public or structured setting.*

*Here is an analysis* of each set of keywords:

1. Topic 18: Reporting and Public Accountability
  *This topic focuses on the communication and assessment aspects of a system or organization.*
  - Keywords: report, public, measur, qualiti, improv, transpar, talk
  - Subject: Public Reporting, Quality Improvement, and Transparency.
  - Context: This cluster suggests an emphasis on creating reports, ensuring quality, making improvements, and maintaining transparency, especially when dealing with the public. It reflects external communication and accountability.
2. Topic 16: Organizational Culture and Focus
  *This topic emphasizes the internal, non-structural elements that drive an organization.*
  - Keywords: organ, focus, cultur
  - Subject: Organizational Culture and Strategic Focus.
  - Context: The keywords clearly point to the internal environment (culture) and the direction or priority (focus) of a body or organization. It deals with the soft elements of management.
3. Topic 21: Strategic Planning and Leadership Development
  *This topic centers on the mechanisms for future action and personnel management.*
  - Keywords: plan, leader, goal, strateg, staff, question, educ, develop, set, process, data
  - Subject: Strategic Planning, Leadership, and Staff Development.
  - Context: This is the most structured topic, covering the full cycle of setting a goal, creating a plan and strategy, managing staff and leadership, and using data and processes. It is heavily focused on future action and human capital.

*Overall Topic Subject*

*The combined analysis points strongly toward a body of text discussing Strategic Management and Governance in a Quality-Focused Organization, likely with a mandate for public accountability* *(e.g., government, non-profit, or a large corporation reporting to stakeholders).*

### 4.2. Factors 2–10

Each of the remaining factors was submitted to Gemini in turn, and the analysis results were captured for each. Table 5 summarizes the AI interpretations for each of the 10 factors. Definitions of the topics are also provided.

### 4.3. A Framework

Gemini continued its analysis voluntarily, providing a framework for context. The researchers did not request the interpretation that follows (bold face added).

*With the addition of Factor 10, the study’s scope on Quality Management is complete, covering the **Structure**, **Process**, and **Outcome** of the healthcare organization, all the way up to Executive Leadership Intervention:*

- *Structure/Roles (F1, F6, F10): Who sets the strategy and handles high-level issues? (Strategy, Learning Foundation, Executive Governance).*
- *Process (F2, F4, F5, F7, F8): How is the work done, managed, and improved? (Clinical Operations, Data Mechanism, Goal Setting, Improvement Cycle, Initiation).*
- *Outcomes (F3, F9): How is success defined and measured? (Performance Metrics, Success/Impact on Patients).*

### 4.4. Graphical Framework

**Figure 1:**
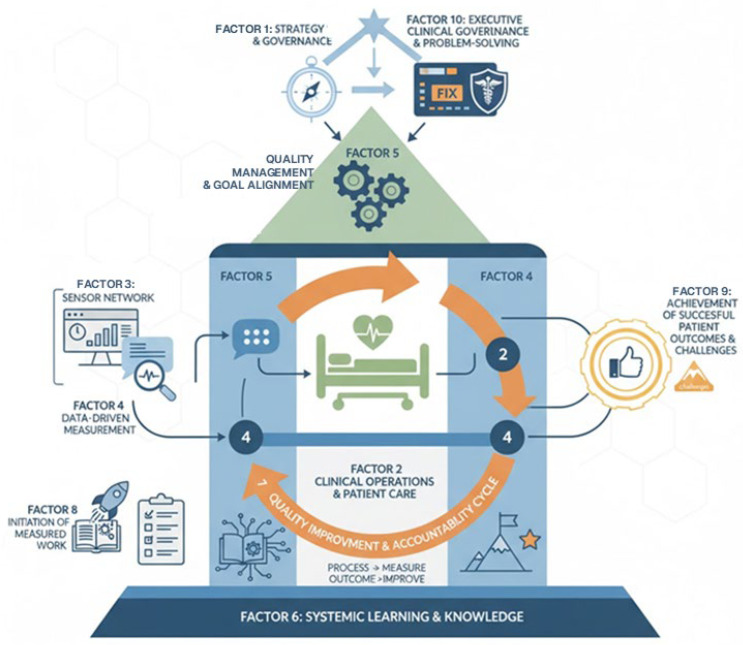
First Graphical Representation of the Factors. (Additional or alternative images appear in the chat session, which is accessible from https://gemini.google.com/share/d66028e6b36a (accessed on 11 January 2026).

Gemini was then asked to draw a graphical representation of the factors, which is included as Figure 1. Its initial response to the request included the following:

*Okay, here’s a graphical model representing the 10 factors you’ve identified, showing their logical connections within a comprehensive framework for Healthcare Quality Management & Performance.*

*I’ve used different colors and icon styles to subtly group them into structural, process-oriented, and outcome-oriented components.*

## 5. Discussion

The objectives of this study were twofold: first, to review the capabilities currently available to the healthcare community by AIs for analyzing qualitative data. The second objective was to explore interviews with chief nursing officers at some of the most successful academic medical centers in the country. The objective of the interviews was to discover what made those hospitals so successful, as perceived by the nursing leaders. To provide illustrative examples of the themes identified in this analysis, Table S1 (see the Supplementary Materials) contains researcher-selected representative quotes for each topic. The researchers selected a representative quote for each topic by carefully reviewing the interview transcript and the LD matrix text generated by the LSA solution.

### 5.1. AI Contribution

Pre-AI, most text mining analysis needed an analyst with a fairly high level of technical skill. The analysis was based entirely on probabilistic and/or algebraic algorithms, meaning the analyst must have a fairly strong math skill set. The analyst also needed an understanding of various natural language processing techniques and the ability to program, usually in Python, R, or possibly Java.

All the AIs reviewed as part of this study had built-in text-mining capabilities. Not all would execute all known methods; for instance, Gemini 2.5 would not run LSA or LDA; however, it would write a Python program to perform LSA or LDA, which could be downloaded and run by the analyst. Yet all of the systems integrate some form of the Microsoft TnT-LLM framework, with variability in how the Taxonomy Generation or the decomposition/clustering stage is executed. In a separate test with a different dataset, left to its own devices, Gemini 2.5 generated a taxonomy by vectorizing a *tf-idf*-weighted matrix, then selecting the number of factors to extract using the elbow method. Finally, it discovered the factors or clusters using a K-means clustering algorithm. This method is generally acceptable for performing text mining [38].

To validate the AI analysis results in Section 4, the researchers independently evaluated the keyword lists provided to the AI and compared them with the labels and descriptions generated by Gemini. Since two AI systems were used, LSA and Gemini, validation also included two researchers independently reviewing samples from the source interview transcripts. The results were then compared, with any disagreements re-examined jointly. The researchers are satisfied that LSA and Gemini 2.5 performed satisfactorily.

The AIs tested handled most of the higher-level tasks that the analyst needed. That is not to say the analyst does not need training, but the level of skills is reduced to a more fundamental knowledge level. In fact, an untrained individual could probably ask the AI to analyze the data to determine the optimal number of topics and then ask the AI to define and describe those topics. All of the AIs knew that text mining analysis requires data cleaning and preprocessing, how to determine the optimal number of topics to extract, and had at least one method for decomposing the data.

In traditional text mining, once the data are decomposed into topic clusters, the terms and source texts that constitute the clusters must be examined manually by extensively reading to define the various topics. This process can be very tedious, bias-prone, and time-consuming. The AI can decompose the data and handle this task in seconds with a very simple script:

*Analyze the data in the attached file. Identify the clusters in the data, then define and describe them.*

The advantages and conveniences that AIs offer the medical community are tremendous. Where a midsized medical system might have had a person reading hundreds of documents occasionally to gain insight into, for example, patient opinion, the analysis can now be performed far more routinely and frequently. Most medical systems will have personnel in their IT departments who can quickly learn to perform this task without additional formal training. The capability also supports other unstructured data studies, enabling management to listen to the voice of the patient and drill down into other datasets.

### 5.2. Modeling Healthcare Success—Donabedian as a Complex System Model

Healthcare, in general, is experiencing unprecedented change. Advancements in scientific know-how, biomedical knowledge, and pharmaceuticals alone are staggering [39]. On top of that, policies, regulations, insurance processes, staff readiness, and value-based purchasing [40] combine to create a highly complex situation for the medical manager to deliver top-quality medical care while maintaining profitability. Nevertheless, several academic medical centers have successfully navigated the environment. The interviews analyzed in this study were intended to identify what made those institutions successful.

The model that emerged from the data and is illustrated in Figure 1 is a variant of the Donabedian model, which is premised on structure, processes, and outcomes [27]. However, that model was not used to develop the interview questions, nor was it mentioned in the interviews or the prompts used in the AI analysis. The attributes of the model appear so well established in those organizations that they are de facto or standard practice. While too subtle for the casual reader to detect, the AI still picked up on the model’s presence.

The Donabedian model naturally and independently emerged from the AIs’ analysis. First, neither the raw corpus nor the X-matrix used in the Section 3.2 analysis was ever exposed to Gemini during the Section 4 analysis. Second, the raw source data, and therefore the X-matrix as well, do not mention the term Donabedian. Third, the analysis in Section 4 was not repeated—the results reported in Section 4 were generated in the first and only run of the analysis and constitute the AI’s first exposure to the factor solutions. Finally, the researchers did not suggest any interpretation at any point. In fact, as reported in Section 4.3, the AI stated, “*With the addition of Factor 10, the study’s scope on Quality Management is complete, covering the Structure, Process, and Outcome …*”.

After generating the graphics for Section 4.4, a final prompt was sent to the AI. It read,

*[A]bove you offer a Final Framework content section in which you define the elements of the framework as consisting of structure/role, process, and outcome. Can you do a literature review of these using the academic literature?*

It was at this point that the AI disclosed the Donabedian model. The full chat session may be accessed at https://gemini.google.com/share/d66028e6b36a.

There appears to be a persistent tendency in the literature to oversimplify Donabedian’s conceptualization of healthcare quality by treating it as a reductionist, linear framework of structure, process, and outcomes rather than as an integrated system [41,42,43,44,45]. Evidence of this fragmented interpretation can be found in McCullough et al.’s 2023 [27] systematic review of primary healthcare nursing service evaluations using the Donabedian model. Their finding that most of the thirty-two reviewed studies focused primarily on outcomes at the exclusion of structures and processes suggests a tendency to fragment what Donabedian intended as an integrated conceptualization [27].

Berwick and Fox emphasize that “Donabedian was far from a reductionist” [46]. In his 1989 article, Berwick argued that measuring quality must include the interplay among structure, process, and outcomes [47]. Donabedian again articulated this integrated conceptualization when reflecting on the failure of American organizations to replicate Japanese quality improvement success, noting that quality is never the product of a single isolated intervention but emerges from “a whole constellation of factors” [48].

The present research provides empirical support for this more complex interpretation of the model. Importantly, the Donabedian model did not guide the study’s design. No interview questions or interview responses explicitly mentioned the Donabedian model. Yet the fundamental dimensions of structure, process, and outcomes emerged naturally from participants’ narratives. This organic emergence strengthens the argument that the Donabedian model captures foundational dimensions of quality that manifest even when they are not explicitly sought.

Further, the statistical findings reinforce this interpretation. Cross-loadings observed in Table 3 among Factors 3, 4, and 6 reveal meaningful empirical overlap across what traditionally might be categorized as outcomes, processes, and structures.

- Outcomes: Factor 3—‘Healthcare Quality Metrics and Data Infrastructure.’
- Processes: Factor 4—‘Interprofessional Communication and Data-Driven Improvement.’
- Structure: Factor 6—‘Systemic Learning and Knowledge Infrastructure.’

Rather than appearing as isolated constructs, these domains demonstrate statistical interdependence. This pattern directly reflects Donabedian’s insistence that quality should be understood as “…an unbroken chain of antecedent means followed by intermediate ends which are themselves the means to still further ends” [24] (p. 694). In these high-performing academic medical centers, the components of structure, process, and outcomes do not function as sequential stages; instead, they operate as simultaneously active, mutually reinforcing dimensions within a single integrated quality system.

Taken together, these findings suggest that quality improvement in successful healthcare organizations is not the product of optimizing isolated elements but instead emerges from dynamic interactions among structures, processes, and outcomes working in continuous concert. What makes this particularly compelling is that this ‘systems level’ interpretation emerged from chief nursing officers’ reflections without being theoretically imposed, indicating that such integration may represent how quality is actually enacted in practice rather than merely how it is theoretically idealized. Accordingly, this study not only challenges the persistent reductionist interpretation of the Donabedian model but also provides empirically grounded insight into how its components function as a living, recursive system within real-world, high-performing healthcare environments. The factors and themes identified here, therefore, offer a meaningful foundation for future work in healthcare quality management and for a renewed conceptualization of Donabedian’s framework as a complex system of feedback, adaptation, and mutual reinforcement rather than a linear evaluative tool.

## 6. Limitations

This work is not without limitations. The data only included observations from chief nursing officers. Input from nursing subdepartment leaders, floor charge nurses, and administrators outside the nursing area could contribute unexpected facets to the model. The data was also collected exclusively from teaching hospitals with COTH membership. Successful proprietary organizations might as well contribute to the model.

Given that the data were collected in COTH member organizations and that the interview questions included specific COTH references, applying the results to nonprofit and governmental hospitals could prove challenging. That said, the main model is grounded in quality improvement (the house of quality) and might prove valuable.

In the analysis, the interview data were clustered with LSA. Sometimes another text mining method (LDA, pLSA, NMF, k-means, etc.) might yield different results; however, given the nature of the sample (CNOs from a small population of COTHs), the likelihood of a radical difference is remote.

### 6.1. Upload Risks

The AI results (Section 4) were obtained exclusively from Gemini 2.5. While the article was being written, Gemini 3.0 was deployed. No attempt was made to run the data in 3.0. Since the Gemini models had not previously been exposed to the data, the AI solutions reported are as pristine as possible. In part, we were concerned about Google’s efforts to improve its models. Per Gemini, “*in the free tier of Gemini, that data [uploaded data] is stored on Google’s secure servers. However, how it is handled depends entirely on your settings.*” The default retention period is 18 months. Further, the data may be used to improve “machine learning technologies,” and samples from the uploaded data could be passed to a human for review. Note that each AI is different. Microsoft’s Copilot reports, “*I don’t personally store your data or keep a memory of it unless you explicitly ask me to remember something*.” So, care must be exercised, particularly if the data is sensitive.

### 6.2. Ethical Considerations

There are ethical considerations with using publicly available AIs. Recent research by Hou et al. (2025) identified nine themes in the healthcare literature regarding AI ethical concerns [11], including privacy protection, algorithmic bias, deterioration of clinical skills, technical reliability, accountability, and data security. Claude.ai provides a helpful outline of considerations for developing a policy regarding uploading data to a commercial AI. That full chat session can be reached using the URL (https://claude.ai/share/fbbb0156-eddb-413c-a3ae-ca703bf624e9, accessed on 5 January 2026). Some of the highlights are presented here.

Core Ethical Principles

- Consent and Privacy: Before uploading any data, ensure you have proper authorization. Consider whether the data contains sensitive information like health records, financial details, or personally identifiable information (PII).
- Data Minimization: Only upload what is necessary for your specific purpose.
- Understanding Terms of Service: Carefully read the AI provider’s terms regarding data handling.

Organizational Considerations

- Internal Policies: Many organizations have established protocols for what data can be shared with third-party services
- Risk Assessment: Evaluate potential harms if the data were to be exposed or misused.
- Intellectual Property: Be cautious about uploading copyrighted materials, trade secrets, or proprietary code that could compromise competitive advantages or violate licensing agreements.

One final question was asked of Gemini regarding ethics and security:

*Are there any AIs available that could be contracted by a healthcare system and installed on a standalone server*

*Yes, numerous AI solutions for healthcare are available for contracting and can be deployed on-premises, within a healthcare system’s secure infrastructure. This approach, often referred to as “private AI” or “edge AI,” is specifically designed to meet stringent data privacy and compliance regulations like HIPAA by ensuring sensitive patient data never leaves the organization’s control.*

## 7. Conclusions

The objectives of this study were to review the capabilities of LLM AI for qualitative analysis and to identify the key factors of success in top-performing academic medical centers. This study demonstrates that freely available LLMs offer a practical approach to qualitative analysis, generating insights that traditionally require significant expertise and time. The application of LLM AI to qualitative interview data with chief nursing officers at top-performing academic medical centers provides evidence of validation of LLM AI utility, yielding a model of organizational excellence consisting of ten factors and twenty-four subtopics.

Additionally, the LLM independently identified a theoretical linkage that manual analysis had missed, the Donabedian structure-process-outcome framework. Providing evidence that these dimensions represent fundamental operational realities rather than theoretical constructs. Furthermore, this finding challenges the pervasive linear interpretations of the Donabedian model, revealing quality as a complex, recursive system where structure, process, and outcomes function as mutually reinforcing dimensions. Quality excellence appears to emerge from the dynamic interplay of interactions rather than sequential optimization of isolated elements.

From a practitioner perspective, LLM AI tools can potentially democratize access to sophisticated qualitative analytics, enabling individuals with basic technical skills to extract actionable insights from large volumes of textual data. This allows organizations to translate text data into actionable insights to drive real, rapid organizational change and improvement.

While promising, this work represents an initial step. Future research should test these methods across broader datasets, explore comparative performance with alternative platforms, examine reliability across analytic conditions, and assess how organizations translate AI-derived insights into practice. Nonetheless, this study provides compelling evidence that LLM AI can meaningfully augment healthcare research and serve as a resource for building knowledge and advancing organizational excellence.

## Figures and Tables

**Table 1:** Interview questions directed to Nursing executives.

1. To what extent does public reporting of patient outcomes data drive quality improvement?
2. What makes your hospital different from other COTH (Council of Teaching Hospitals) member hospitals? What does this hospital have that other hospitals do not?
3. Is a specific approach to organizational change being used in this organization? If so, please describe it.
4. What strategies are used to identify future threats and opportunities?
5. At what point did your organization come to the realization that connecting clinical quality outcomes to compensation models, as exemplified by Value-Based Purchasing, would become the new standard? What triggered that realization?
6. At what point did your organization begin tracking the specific measures used in Value-Based Purchasing? What triggered you to do so? How did these measures differ from what you have tracked in the past?
7. How are goals developed at this organization? What role do your health care system, governing board, executive leadership, physicians, nurses, staff, and patients have in the formulation of goal development? What is your goal(s) regarding Value-Based Purchasing measures?
8. What were the major challenges with sorting through and figuring out where your organization stood based on the Value-Based Purchasing metric?—How were they overcome?
9. What role do physicians play in quality enhancement, performance improvement, managerial decision making, cost-cutting activities, etc.?
10. How are specific goals communicated throughout the organization?
11. How are specific goals translated into practice? What are common challenges, how are they overcome—Example?
12. How are successes and failures addressed and communicated throughout the organization?
13. How is resistance to change overcome in your organization?
14. How is the organization implementing/enhancing a culture of quality care? How is the hospital’s administrative and physician relationship developed to enhance this culture?
15. What technologies or techniques have you adopted/adapted to measure, monitor, and improve Value-Based Purchasing measures? How is this information being used and distributed throughout the organization?
16. What has been challenging with monitoring your performance regarding VBP?—How have these challenges been overcome?
17. How are the ideas and concerns of front-line employees being communicated to leadership?
18. Are there any reward systems in place to motivate front-line employees to contribute toward continuous improvement?
19. How would you describe this hospital’s organizational structure? What role does organizational structure play in Value-Based Purchasing performance?
20. Please define, in your own words, what high performance is at this institution. What are the top 5 factors that are driving high performance at this institution?
21. What are the three most important attributes of a successful hospital leader?
22. Where will your hospital be in 5 years?
23. Are there any questions that I have not asked but that you feel I should have? (What would you want to ask these hospitals’ leaders?)

**Table 2:** LSA Term (LT) Solution Matrix—first 10 and last 3 topics. Color in topic headings provides a connection with Table 3’s factor solution *.

Topic 1	Topic 2	Topic 3	Topic 4	Topic 5	Topic 6	Topic 7	Topic 8	Topic 9	Topic 10	…	Topic 22	Topic 23	Topic 24
improv	patient	system	goal	nurs	hospit	process	good	care	data		score	communic	start
measur	safeti	health	qualiti	physician	nurs	measur	stuff	provid	understand		hospit	goal	work
process	qualiti	data	measur	leadership	medic	outcom	work	patient	transpar		depart	leadership	talk
qualiti	nurs		improv	hospit	chief	staff	pretti		perform		patient	qualiti	measur
	committe		relat	role	health		differ		chang		nurs	specif	process
			perform	clinic	system				creat			staff	metric
			depart	tabl	care				metric				
				administr	clinic				tool				
				work					staff				
									good				

* The elements reported in this table are the roots of the words/terms. Improving, improves, and improved are variants of the same root.

**Table 4:** Topic Solutions Reorganized by Factor.

Factor 1			Factor 2			Factor 3			Factor 4		
Topic 18	Topic 16	Topic 21	Topic 9	Topic 2	Topic 15	Topic 22	Topic 3	Topic 14	Topic 10	Topic 12	Topic 5
report	organ	plan	care	patient	line	score	system	team	data	talk	nurs
public	focus	leader	provid	safeti	day	hospit	health	nurs	understand	meet	physician
measur	qualiti	goal	patient	qualiti	unit	depart	data	servic	transpar	nurs	leadership
qualiti	cultur	strateg		nurs	servic	patient		perform	perform	differ	hospit
improv		staff		committe	patient	nurs		come	chang	tell	role
transpar		question			exampl			abl	creat	issu	clinic
talk		educ			talk			question	metric	staff	tabl
		develop						purchas	tool	tabl	administr
		set							staff		work
		process							good		
		data									
**Factor 5**			**Factor 6**	**Factor 7**			**Factor 8**		**Factor 9**	**Factor 10**	
**Topic 4**	**Topic 23**	**Topic 11**	**Topic 19**	**Topic 17**	**Topic 7**	**Topic 1**	**Topic 24**	**Topic 8**	**Topic 13**	**Topic 6**	**Topic 20**
goal	communic	manag	level	happen	process	improv	start	good	challeng	hospit	call
qualiti	goal	chang	term	process	measur	measur	work	stuff	work	nurs	issu
measur	leadership	staff	learn	come	outcom	process	talk	work	stuff	medic	caus
improv	qualiti	qualiti	system		staff	qualiti	measur	pretti	hospit	chief	work
relat	specif						process	differ	patient	health	fix
perform	staff						metric		success	system	
depart									impact	care	
										clinic	

**Table 5:** AI Interpretations.

Factor 1 Strategic Management and Governance
Topic 18: Reporting and Public Accountability
Topic 16: Organizational Culture and Focus
Topic 21: Strategic Planning and Leadership Development
Factor 2 Clinical Service Delivery and Quality Assurance
Topic 9: Care Provision and Patient Focus
Topic 2: Safety, Quality, and Clinical Governance
Topic 15: Operational and Unit-Level Service
Factor 3 Healthcare Quality Metrics and Data Infrastructure
Topic 22: Performance Metrics and Hospital Structure
Topic 3: Data Management and Health Systems
Topic 14: Service Performance and Team Effectiveness
Factor 4 Interprofessional Communication and Data-Driven Improvement.
Topic 10: Performance Data and Organizational Change
Topic 12: Communication and Discussion Forums
Topic 5: Clinical Leadership and Professional Roles
Factor 5 Quality Management and Goal Alignment
Topic 4: Quality Improvement and Performance Measurement
Topic 23: Communication and Leadership for Quality Goals
Topic 11: Management of Quality and Change
Factor 6 Systemic Learning and Knowledge Infrastructure
Topic 19: Systemic Learning and Terminology
Factor 7 Quality Improvement and Accountability Cycle.
Topic 17: Events and Processes
Topic 7: Outcome Measurement and Staff Involvement
Topic 1: Improvement Cycle
Factor 8 Initiation of Measured Work and Informal Assessment
Topic 24: Initiating Work and Process Metrics
Topic 8: General/Qualitative Assessment and Work
Factor 9 Achievement of Successful Patient Outcomes
Topic 13: The Impact and Difficulty of Hospital Work
Factor 10 Executive Clinical Governance and Systemic Problem-Solving
Topic 6: System and Clinical Leadership
Topic 20: Problem Identification and Resolution

## Data Availability

The datasets presented in this article are not readily available because the data are part of an ongoing study. Requests to access the datasets should be directed to S.C.

## Supplementary Materials

The following supporting information can be downloaded at: https://www.mdpi.com/article/10.3390/healthcare14020248/s1, Table S1: Factors, Topics, and Researcher Identified Representative Quotes.

## Abbreviations

The following abbreviations are used in this manuscript:

AI	Artificial Intelligence
BoW	Bag-of-Words
CMS	Centers for Medicare and Medicaid Services
CNO	Chief Nursing Officer
COTH	Council of Teaching Hospitals
IRB	Institutional Review Board
LDA	Latent Dirichlet Allocation
LD	Loadings for Documents
LLM	Large Language Model
LSA	Latent Semantic Analysis
LT	Loadings for Terms
NMF	Non-negative Matrix Factorization
pLSA	Probabilistic Latent Semantic Analysis
SVD	Singular Value Decomposition
TF-IDF	Term Frequency-Inverse Document Frequency
VBP	Value-Based Purchasing
