# Supplementary material for: AI-Enhanced Qualitative Analysis in Healthcare: Unlocking Insight from Interviews of Leadership at Top-Performing Academic Medical Centers
Source: Healthcare (Basel). 2026 Jan 19;14(2):248. doi: 10.3390/healthcare14020248 (PMC12841440; doi:10.3390/healthcare14020248)
Supplement: Supplementary file 1 [file healthcare-14-00248-s001.zip › healthcare-4068442-supplementary.pdf]

Table S1 - Factors, Topics, and Researcher Identified Representative Quotes		
**Factor**	**Topic**	**Representative Quote**
1-Strategic Management and Governance	18-Reporting and Public Accountability	We are proud to be transparent, obviously, ‘cause we had great results, but people – the public are making decisions based on publicly reported data.
	16-Organizational Culture and Focus	When we found that there is something new that we didn’t have, what we tried to do is be proactive. So we have teams in performance services who read the federal register, so they know what’s coming forward. We have teams that work with the joint commission to be able to say, okay, the joint commission is expecting this in the future.
	21-Strategic Planning and Leadership Development	Do different constituencies play a major role in goal development? Do the physicians play a major role, the board, the executive staff? We have a fairly diverse process. So in the strategic planning process of the health system we had inter-professional teams around clinical practice, around research, around education. So physicians, nurses, faculty, administrators were all in those diverse groups together to plan. And when our EDPMA sort of updates the organization on the plan everyone is invited to hear and to provide feedback so we have a fairly collaborative process around that. So, yes, physicians are involved, nurses are involved. Everybody has the opportunity to understand what they are.
2 - Clinical Service Delivery and Quality Assurance	9-Care Provision and Patient Focus	Decision making closest to the work is really a founding principle as far as I’m concerned. We really cannot understand the experience of a physician at the bedside, a nurse at the bedside, a patient or family member without engaging them if that’s the issue we’re trying to solve.
	2-Safety, Quality, and Clinical Governance	I’ve always really allowed the units to focus on their goals for their own. So, say patient satisfaction. If you have a med surg floor, you’re in OR or you have an oncology floor, the goals for patient satisfaction will be similar for the most part across the board, but, you know, there’s going to be nuances that are very specific to that patient population. And I think it’s very important for those people who take care of those patients to identify what are they?

Table S1 - Factors, Topics, and Researcher Identified Representative Quotes		
	15-Operational and Unit-Level Service	And so, I've taken to translating that by unit to, "How many days have we gone without a central line infection?" "How many lives have we saved if we don't have any ventilator associated pneumonia?" "And so, if our mortality rate goes from 1.2 to .75, how many lives did we save?"
3 - Healthcare Quality Metrics and Data Infrastructure	22-Performance Metrics and Hospital Structure	They are really – you know, so, I sent them – now, we have this little program that we can send a recognition and give them points. So, I did that to Ryan because he's totally owning the whole imaging department and there's about, you know, six or eight of them that have individualized scores. And I'm like, "I can't wait to show you this at the next meeting. We're going to show everybody. Look at your scores. They're fabulous", you know? But, they know it's not – the scores aren't just for the sake of the scores. That's the other piece. And I really do try to bring it back to, "Why are we doing this?"
	3-Data Management and Health Systems	I think it helps actually drive quality because again, being part of a system, we're able to benchmark internally fairly quickly. We're able to disseminate best practice across the system.
	14-Service Performance and Team Effectiveness	That's how I would see it. I think about high performance in terms of team functioning, true team functioning in collaboration. For me it's never a numerical target that one is supposed to hit or exceed, no. It's bigger than that.
4 - Interprofessional Communication and Data-Driven Improvement.	10-Performance Data and Organizational Change	Data, data, data, data. Is the data right? Is the data current? Are the data definitions right? Do we believe the data? So, that's – I mean, that's huge. And then data transparency. So, you get the data. You finally extract it, excavate it, and then it's not very timely. It's – you know, you get a lot of pushback initially from people about the data's not right. Then, you go through, you know, iteration after iteration of how do you help people get to a place where they feel really comfortable with being transparent with the data
	12-Communication and Discussion Forums	They need to be part of the solution and be at the table for the discussions. So, we'll share. We'll meet with them and we'll share with them some of the costs contained, you know, attributed to the expenses. We'll look at the budgets, you know? We talk about it a lot. We talk about it at the administrative level. We – XXX and I and YYY,

Table S1 - Factors, Topics, and Researcher Identified Representative Quotes		
		financial, we all attend the MAC, which is a requirement, but we're part of that meeting as well. We may not be a voting member, but our physicians bring things to the table there that we can speak to. And if I can't speak to it, one of us can speak to that challenge. And if we then need to, if it's bigger than just that discussion, then we follow up with meeting with them offline and discuss it, you know? And so, things escalate, but then you have to just bring them back and bring them to the table to talk about it.
	5-Clinical Leadership and Professional Roles	I think there is a number of things. One thing that I think has been a key contributor to our success is what we refer to as a paired leadership model, where we've got physician leadership, nursing leadership and administrative leadership working in concert with one another.
5 - Quality Management and Goal Alignment	4-Quality Improvement and Performance Measurement	He said this as, "I'm putting' the stretch goal up here – five years," and that was like way out there, for someone to say, "We're going' to zero; no harm."
	23-Communication and Leadership for Quality Goals	I think the leadership from the top down is not willing to accept mediocrity and that is communicated from the time you're interviewing and as you're stepping into an organization. It's communicated. We expect you to excel. So, if you're not in the green, what are you doing to get in the green? So, it's that constant driving the performance.
	11-Management of Quality and Change	I don't honestly think it matters what change management tools you use. I think the key is that we've put our entirely leadership team down to the manager level, and even some staff, through change management classes that involve discussion and role playing, and I think that really – because I don't know if one method's better than another, but that everybody's on the same page and speaking the same language
6 - Systemic Learning and Knowledge Infrastructure	19-Systemic Learning and Terminology	And then we try to figure out where else could this mistake happen, and then we have a – we communicate it broadly, at the senior level, at the director level, in physician meetings, wherever pertinent, so that people can translate that learning across the system. So we're doing – I think we're doing a much better job – we don't hide from

Table S1 - Factors, Topics, and Researcher Identified Representative Quotes		
		mistakes that we make. We used to. But we don't any longer. We say, let's talk about something bad that happened here, at ...... Medical Center, and how can we learn from it? So we don't – we don't hide from it.
7 - Quality Improvement and Accountability Cycle.	17-Events and Processes	We did central line rounds, for example, as a leadership team and went out and looked at lines and talked to staff about what are your barriers to making this happen? Every time we had an infection we had a mini event analysis to say what happened? Why did this happen? How can we learn from this? So, it's not perfect, but again, we've tried to instead of managing to the numbers we've tried to figure out what are the processes we need to put in place to make this stuff happen? And then build the workflow so that they happen, so that the process is placed.
	7-Outcome Measurement and Staff Involvement	So, for instance, if it's a peri-op, it happens in the operating room, you know, I might select somebody in that area. If it's the first-day, post-op glucose level in critical care, I need a process owner in critical care. If it's a second-day glucose level, I need a process owner on the tele floor. You know, so I need to line all of those up and get the people accountable for each one of those.
	1-Improvement Cycle	And just as a metric of number one, the amount of improvement work that's going on, but also the acceptance that they don't have to be big, grandiose things. And many of them weren't necessarily on your AOP (annual operating Plan) – it could have been, but not necessarily. But it was a reflection, I think, of how pervasive that – just that improvement methodology in work is happening, in terms of our cultural shifts.
8 - Initiation of Measured Work and Informal Assessment	24-Initiating Work and Process Metrics	Because when we first started, when we first started, we sent these metrics out, and people are, "What do you want me to do with this? You know, first of all, they're a month old. I don't know why they failed. I don't recall the case well enough. I don't own that. You know, you're talking about critical care, I work in the operating room." So, we had to get way more deliberate and accountable for that.
	8-General/Qualitative Assessment and Work	Clear communication and transparency of information; good, bad, and ugly, the good, the bad, and the ugly as I say it.

Table S1 - Factors, Topics, and Researcher Identified Representative Quotes		
9 - Achievement of Successful Patient Outcomes	13-The Impact and Difficulty of Hospital Work	I think the most common challenge is to position the goal in a way that helps people have a line of sight to it. So if it's very high level and you're a nurse working every day on a unit, generating margin, for instance, isn't clearly understood. They don't know what they're daily work is supposed to be doing to generate margin. So those kind of line of sight issues are a challenge. It requires us to help people understand why we have that goal in the health system and what it means for them.
10 - Executive Clinical Governance and Systemic Problem-Solving	6-System and Clinical Leadership	Not just hospitals, but children's services, behavioral health and psychiatry services and the adult enterprise. That structure is very important to not having silos about geography, hospital clinic, hospital clinic. And then, my position as the executive for nursing is across the whole of the health systems. So, there are chief nursing officers for the entities like the hospitals and the clinics, but they all report to me to maintain a strategy for across the continuum. So, that piece of the structure is really important. We also have a very – and I mentioned this before – a very strong commitment to paired leadership. So, the Heart Institute, as an example, is led by a nurse and a physician.
	20-Problem Identification and Resolution	We actually have what's called a "in-touch" line that those calls, if you have a concern, they go to an outside organization. That outside organization translates and sends that message back to us so that there is no fear. So we have multiple ways that people can provide that feedback.
